# Spherical GTM: A New Proposition for Visualization of Chemical Data

**DOI:** 10.1002/minf.202500045

**Published:** 2025-06-16

**Authors:** Farah Asgarkhanova, Marcou Gilles, Mikhail Volkov, Murielle Muzard, Richard Plantier‐Royon, Caroline Rémond, Dragos Horvath, Alexandre Varnek

**Affiliations:** ^1^ Laboratory of Chemoinformatics UMR 7140 University of Strasbourg Strasbourg France; ^2^ Université de Reims Champagne‐Ardenne CNRS ICMR Reims France; ^3^ Université de Reims Champagne‐Ardenne INRAE FARE UMR 614 AFERE Reims France

**Keywords:** data visualization, manifold learning, Spherical Generative Topographic Map

## Abstract

The Spherical Generative Topographic Mapping (SGTM) method represents an intuitive approach to visualize chemical data. Unlike the original Generative Topographic Mapping algorithm, which utilizes a bounded flat Euclidean space as a manifold, our proposed modification introduces a spherical manifold to address known nonflat topology issues. In this study, we describe the mathematical formalism of this new approach and showcase its ability to visualize 2D electron density patterns of water and benzene and the CosMoPoly chemical library—an enumeration of synthetically accessible molecules. By comparing the outcomes with established references, it is demonstrated that SGTM emerges as a novel 3D data visualization method, offering improved accuracy in the depiction of chemical structures.

## Graphical abstract

The Spherical Generative Topographic Mapping method extends the Generative Topographic Mapping method to a spherical manifold to address known nonflat topology issues. The mathematical formalism of this new approach is described, and its ability to visualize in 2D electron density patterns and a tangible chemical library is showcased.

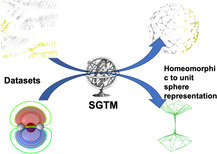

## 1 Introduction

Generative Topographic Mapping (GTM) is a probabilistic, unsupervised machine learning algorithm designed for nonlinear dimensionality reduction and data visualization. It maps high‐dimensional data onto a 2D latent representation while preserving the topological structure of the original data [1]. However, this approach is based on a bounded bidimensional manifold, a geometric shape which may not match the intrinsic topology of a modeled dataset.

Addressing this limitation, Gasteiger et al. applied topological feature maps through a Kohonen neural network (also termed Self‐Organizing Maps) [2] to visualize molecular electrostatic potentials in a 2D format, preserving the topological and geometric features of molecular surfaces. An SGTM‐related algorithm, Spherical‐Principal Probabilistic Surfaces (S‐PPS), was proposed previously by Chang et al [3, 4, 5]. It was applied by Staiano et al [6, 7, 8]. to several data mining contexts, but only anecdotally to chemoinformatics problems. Probabilistic Principal Surfaces (PPS) is an algorithm introduced by Chang et al. in 2001 [9] closely related to GTM. The main difference resides in the metric used to compute the distance of an instance to the manifold. In the GTM algorithm, the distance is estimated at predefined sample points of the manifold called “nodes,” and this distance is isotropic. In the PPS algorithm, the distance of a data point to the manifold is divided into two contributions: one is normal to the manifold, and the other is parallel to it. These contributions can be weighted independently. If the weights are equal, the PPS is equivalent to a GTM.

In this work, we present a modification of the GTM algorithm that utilizes a spherical surface instead of a bounded 2D manifold to fit the data. This adjustment is likely to be more relevant in addressing datasets with inherent spherical topology. The Spherical GTM (SGTM) is illustrated on (1) an intuitively accessible use case for visualization of electron densities and molecular orbitals and (2) chemical space analysis. The term “chemical space” is typically used in two different ways: it may refer to a collection of plausible molecules, or alternatively to the multidimensional descriptor space capturing their structural and functional properties, as well as defining their interrelationships [10]. First, the SGTM visualizing method is illustrated on well‐known compounds—water and benzene—whose well‐established visual characteristics, such as nodal structures, symmetry, and shape of their molecular orbitals, allow for validation of the accuracy and reliability of the algorithm through direct comparison with known references. These examples, with their clear prior expectations, highlight the strengths and limitations of our approach. Second, the GTM and SGTM are compared for the visualization of a tangible chemical space named CosMoPoly—a dataset of synthetically accessible molecules composed via a systematic enumeration of building blocks considering a set of chemical reaction rules. Here, a tangible chemical space is in contrast to a generic chemical space that may include chemical structures independently from their ready synthetic accessibility. The ready synthetic accessibility implies, in this context, an intended chemical synthesis process.

## 2 Experimental Section

### 2.1 Math behind SGTM

The GTM algorithm models the data using a normal distribution of variance *β* centered on a manifold. In the present case, the manifold is a 2D surface of spherical topology: a topological 3‐sphere. This is achieved by the construction of a function |y(u)⟩ where *u* is a location on a 3‐sphere using polar (*θ*) and azimuthal (*ϕ*) angles. The function |y(u)⟩ outputs are *D* dimensional vectors of the input space of the problem. This function is composed of a weighted combination of *L* Gaussian Radial Basis Functions (RBF) centered on pre‐defined locations of the 3‐sphere.

(1) $$\left|y(u)\rangle =W|\Phi_{\mathrm{RBF}}(u)\right\rangle$$

The weights are stored in the D×L matrix *W*, and the *L*‐dimensional column vector $\left|\Phi_{\mathrm{RBF}}(u)\right\rangle$ components are the evaluation of the *L* above mentioned RBFs at point *u*. All RBFs share the same width *σ*.

As in the classical GTM algorithm, and without loss of generality, the probability of the data point $\left|y_{i}\right\rangle$ is proportional to a sum of point evaluation of the density of the normal distribution, at pre‐defined locations of the manifold (nodes).

(2) $$P(|\;y_{i}\rangle |W,\beta )\propto \sum_{k\;\in \;\mathrm{Nodes}}\text{exp}(-\beta \|{\left.\left|y_{i}\rangle -|y(u_{k})\rangle \right\|\right.}^{2})$$

In this expression, the symbol ∥·∥ stands for the Euclidean distance in the *D*‐dimensional input space. Using the estimated probability of each data point of a given dataset, the logarithmic probability of the whole dataset is computed, which is termed “likelihood” in the rest of the article.

Then, the GTM uses an expectation‐maximization scheme to maximize the likelihood of a dataset, optimizing the shape of the manifold (the weights *W*) and the width of the distribution (*β*) [11].

Using polar coordinates for the manifold, however, requires adapting the definition of the RBF to use geodesic distances instead of Euclidean distances [12].

(3) $$\Phi_{\text{RBF}}\left(u\right)\propto \exp-\frac{\delta \theta^{2}}{2\sigma^{2}}$$

where δθ is the angle between the center of the RBF and the point *u*.

Another difficulty is that there are only 5 regular grids on a 3‐sphere. Therefore, the locations of RBF centers and nodes haves to be defined using a quasi‐regular grid. For this purpose, a Fibonacci generalized spiral on a sphere is used [13, 14]. The polar and azimuth angles of the *i*th node of this grid are given by the following equations.

(4) $$\begin{array}{l} \theta_{i}=\arccos\;\left(1-2\;\times \frac{i}{N}\right)\\ \phi_{i}=2\pi \cdot \text{frac}\;\left[\left(\frac{\sqrt{5}+1}{2}\right)\times i\right] \end{array}$$

In these equations, the total number of nodes is *N* and the index *i* is in the range {1..N}.

The last difficulty is the initialization of the manifold. In analogy to the classical GTM, the weight matrix is initialized to fit an ellipsoid. The ellipsoid has the three main axes oriented along the three first principal components of the dataset. Considering the corresponding three first eigenvalues $\lambda_{1}$, $\lambda_{2}$, and $\lambda_{3}$, the lengths of the axis are $\lambda_{3}/\lambda_{1}$, $\lambda_{2}/\lambda_{1}$ and 1.

Finally, the SGTM differs from GTM only by the geometrical shape to define the probability distribution. Therefore, when used on the same dataset, the likelihood (resulting from Equation (2)) of SGTM are comparable to those obtained with a GTM.

### 2.2 Bijection between Two Surfaces

The SGTM builds a map between two surfaces that are abusively termed map and manifold, to be consistent with the terminology of the original GTM algorithm [11]. The first surface is a 3D unit sphere which is the support of the mapping. This means that data points and responsibilities are, in fine, visualized and located on it. The second surface is the center of the normal probability density explaining the dataset. It is a 3D shape, topologically equivalent to a sphere, embedded into the data space and deformed to adjust to the data. In the specific use case (see Section 2.4.1) when the dataset is composed of 3D coordinates, this surface is also a 3D object. This allows to follow directly how the model adjusts to the data.

Hereafter, the unit sphere surface is termed the “map surface,” and the embedded surface is termed a “latent surface” or the “manifold.”

### 2.3 Likelihood Optimizsation Algorithm

As explained above, fitting the SGTM model maximizes the likelihood of the frame dataset. A larger likelihood indicates a better fit. SGTM has 3 tunable parameters, given below in order of importance.

1. Number of Radial Basis Function (RBF) centeres, *m*: number of degrees of freedom of the model. A higher number of RBF centers can capture more complex patterns in the data but may also lead to overfitting;
2. Regularization coefficient, *l*: it controls the expressivity of the manifold. The assumption is that simpler models generalize better to unseen data;
3. RBF width, *w*: the width of the RBFs determines the spread of the RBF used in the model. A larger width results in smoother manifolds, while a smaller width allows for fitting more details in the data distribution.

**Figure 1:**
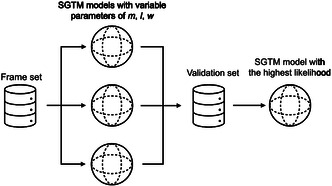
The SGTM model development. A frame set is used to build SGTM models with varying parameters: RBF centers, *m*, regularization coefficient, *l*, and RBF width, *w*. These parameter values are selected to maximize the likelihood on both validation and frame sets.

The SGTM model is built on a frame set. Then, a SGTM model obtained for a given set of parameters’ values (number and width of the RBFs, regularization) is monitored on a validation set. The aim of this exploration is to illustrate how modifications of the parameters’ values impact the likelihood on the validation set: better parameters set produces larger likelihood values on both the frame set and the validation set. Details about this procedure are provided in Supporting Information (Figure 1).

### 2.4 Case Studies

The SGTM is illustrated on two test cases. The first one aims at modeling the data point cloud sampling 3D positions of electrons from the density and molecular orbitals of molecules. The second aims to represent a dataset of Lewis chemical structures systematically enumerated as products of a set of organic reactions and a set of building blocks—a tangible chemical space.

The methodology of the first case study is detailed in Section 2.4.1. The examples, presented herein, with their clear prior expectations, allow us to explore how variations in the SGTM free parameters impact the resulting visualizations.

The Section 2.4.2 presents the methodology for studying tangible chemical space. This use case provides a comparison between the SGTM and the classical GTM equivalent illustrating some situations where the former can be advantageous. This section details the tangible chemical space itself, the settings of the free parameters of SGTM and GTM, and the quality measures used.

These test cases highlight the strengths and limitations of this approach.

#### 2.4.1 Visualization of the Molecular Electronic Densities and Orbitals

The SGTM visualizing method is illustrated on water and benzene.

##### 2.4.1.1 Ab Initio Electronic Structure Calculations

For each compound studied, we computed the electronic densities, Highest Occupied Molecular Orbital (HOMO), and Lowest Unoccupied Molecular Orbital (LUMO). These functions were sampled on a 3D cubic grid.

All calculations have been performed using the ORCA toolkit (v. 4.2.1) and Density Functional Theory (DFT) with the B3LYP hybrid functional and the Karlsruhe DEF2‐SVP basis set [15]. Symmetry considerations were applied where relevant during dataset generation. The detailed methodology is provided in the Supporting Information.

##### 2.4.1.2 SGTM Tunable Parameters

A cloud of 20,000 points mimicking electron density for each selected orbital was visualized, by projection on its optimally fitted (maximal likelihood) manifolds, at varying setup parameters. The number of RBF centers, *m*, varied from 20 to 125, the regularization coefficient, *l*, from 0.3 to 100, and the RBF width, *w*, from 0.1 to 1, generating different models to show how the mapped output depends on the parametrization.

##### 2.4.1.3 Visualizsation Tools

Electron density, HOMO, and LUMO for all molecules were visualized using the MOE software (version 2024.06), [16] with input files generated by SGTM.

The procedure is exemplified on our web server through two services, one for modeling the electronic densities (https://chematlas.chimie.unistra.fr/WebTools/sgtm_ed.php) and the second for molecular orbitals (https://chematlas.chimie.unistra.fr/WebTools/sgtm_mo.php). These services regenerate datasets equivalent to those in this work.

#### 2.4.2 Analysis of Tangible Chemical Dataspace

In this section, we apply the SGTM method to a chemoinformatics problem: visualizing tangible chemical dataspace CosMoPoly. We aim to compare the visualization of the density landscape achieved by GTM and SGTM for the dataset.

This library was preferred in this study over public libraries, as it provides a well‐defined and structurally homogeneous dataset generated through controlled enumeration of building blocks using specific reaction rules. This design enables an evaluation of SGTM's ability to capture subtle structural variations in a library. An analogous study of Tox21 [17] dataset is available in supporting information.

##### 2.4.2.1 Data and Descriptors

To design a tangible chemical library CosMoPoly, 49 Building Blocks (BBs) (the BB list is provided in SI) were systematically combined using two predefined reaction rules (acetalization followed by esterification, and esterification alone, see SI) rules. The enumeration was performed using the Synt‐On software version 2024 [18]. The design molecules featured scaffolds such as carbohydrate linked to polyol and then to organic acid and esters (see section 3.2.1).

The resulting 25,289 molecular structures were encoded using ISIDA Property‐Labelled Fragment Descriptors [19]. The descriptor set used was atom‐centered fragments, with a fragment radius of 2–3 atoms.

The CosMoPoly library is currently being synthesized in part. Being an enumerated chemical library, it shows enough homogeneity to illustrate the potential of GTM and SGTM to locate on the maps chemical structures sharing common scaffolds. This feature is useful to illustrate the comparison between the GTM and SGTM.

##### 2.4.2.2 SGTM and GTM Setup

The GTM was built using 50 RBF (*m*), the regularization (*l*), and the RBF width (*w*) value were left to default. Data centering was applied. These values have been set following a procedure aiming at optimizing the likelihood on frame and validation sets. In detail, the dataset was divided into these two subsets: 12,645 molecules for frame and 12,644 molecules for validation datasets. The approach followed is analogous to the methods described above in section 2.3. For consistency, the SGTM parameters were kept the same to be able to directly compare the outputs.

### 2.5 Spherical Generative Topographic Map Outputs

The SGTM generates three main outputs. The spherical manifold is one of them. It is a 2D geometric object embedded in the data space.

Another output is the unit sphere to which the spherical manifold is mapped. Each data point appears on this sphere as a pattern of responsibilities. It is convenient to compute the center of mass of these patterns. As a result, each data point is resumed to a single point on the unit sphere.

Finally, the unit sphere can be difficult to picture as a 2D image. In this work, we apply a rectangular projection using the polar angle as the *y*‐axis and the azimuthal angle as the *x*‐axis. The reference frame results from the initial alignment of the spherical manifold to the three first principal components of the dataset.

## 3 Results and Discussions

### 3.1 Visualization of the Molecular Electronic Densities and Orbitals

We have chosen two compounds to illustrate the method: water and benzene. Water molecule is well documented (the first quantum chemistry model was published in 1985 [20]) and intuitive, thus facilitating the interpretation and the visualization of results generated by SGTM. Similarly, benzene was chosen due to its distinct molecular structure, extended and recognizable densities and orbital; it presents a slightly more complex and larger problem to illustrate the method.

#### 3.1.1 Parameter Exploration

**Figure 2:**
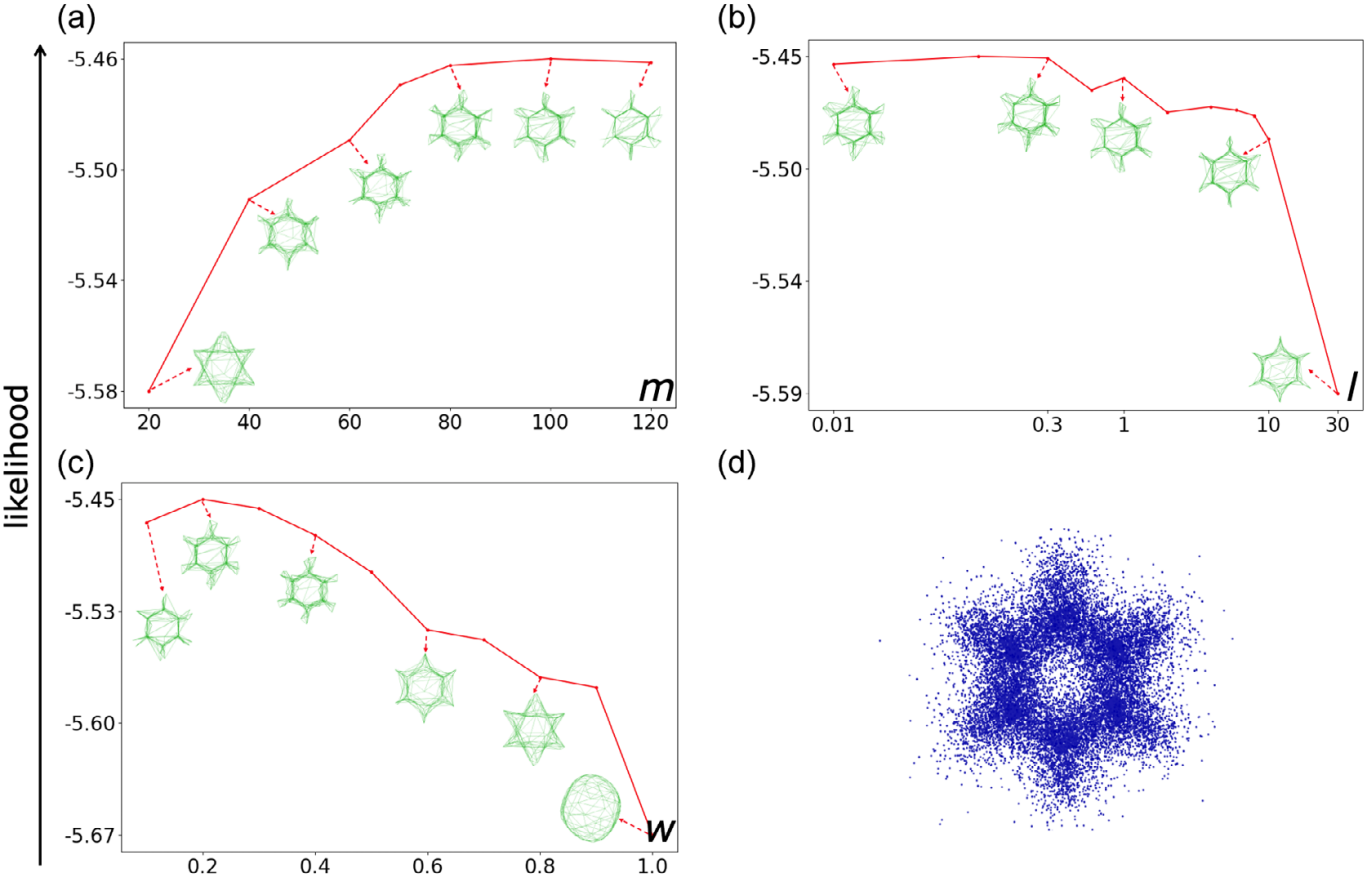
Evolution of the manifold visualization for benzene electron density as a function of SGTM free parameter variations. The likelihoods are recorded on the frame set and the manifolds. The manifolds are visualized with mesh representation. (a) Visualizations of the manifolds varying the number *m* of RBF (*l* = *1*; *w* = 0.2). At *m *= 20, the manifold forms a star‐shaped structure that does not fully represent the electronic distribution of the benzene molecule. Conversely, at *m *= 120, the manifold more closely aligns with the atoms, with some nodes pointing outward toward denser regions in the frame set. As expected, a larger *m* value improves the model's capacity to capture more complex structures. (b) Effect of the regularization parameter, the *l* value (*m *= 80; *w *= 0.2). When *l* exceeds 1, the visualization becomes less detailed, capturing fewer molecular features. (c) Impact of the RBF width, the *w* parameter value (*m *= 80; *l *= 0.3). Large values (*w* > 0.8) result in a smoother visualization, while smaller values (*w* < 0.2) lead to more detailed representations. (d) The frame set is the electron density of benzene, where each electron is represented as a blue dot.

**Figure 3:**
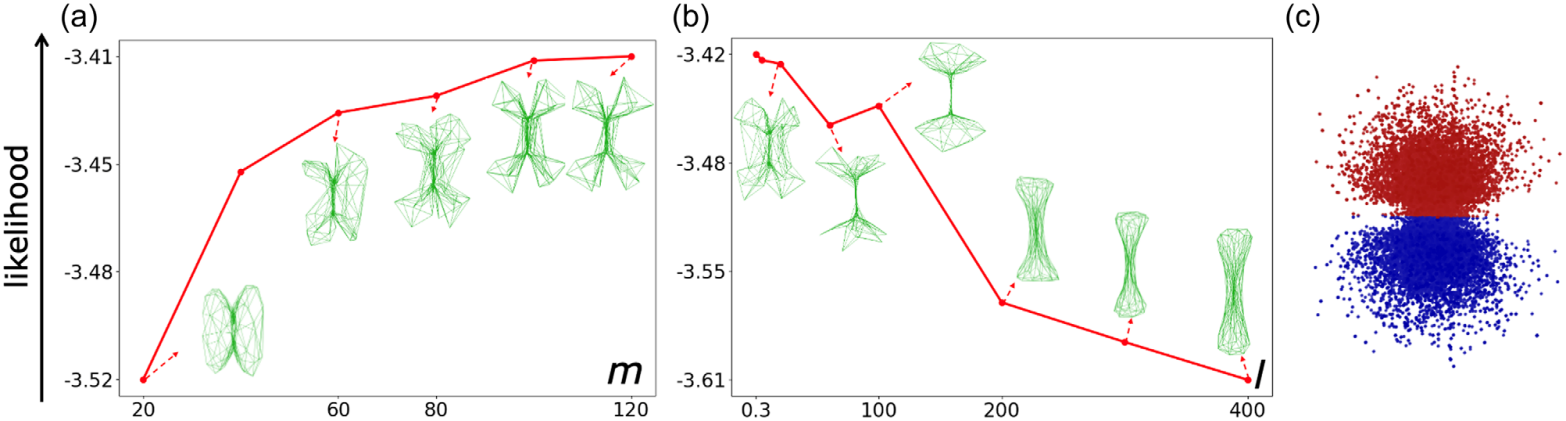
Evolution of the manifold visualization for water HOMO as a function of parameter variations. The *y*‐axis is the likelihood of the frame set. (a) Effect of the number of RBF centers, the parameter m value (*l *= 1; *w *= 0.2). To minimize modeling errors, the manifold needs to be flexible enough to fit the water HOMO nodal plane where the wave function is zero. (b) Effect of the regularization, the *l* parameter value (*m *= 80; *w *= 0.2). At large values, the shape of the manifold simplifies and stabilizes, with fewer changes in its representation. (c) The frame set is the HOMO of a water molecule, where each electron is visually depicted by red and blue dots to illustrate the positive and negative lobes, respectively.

**Figure 4:**
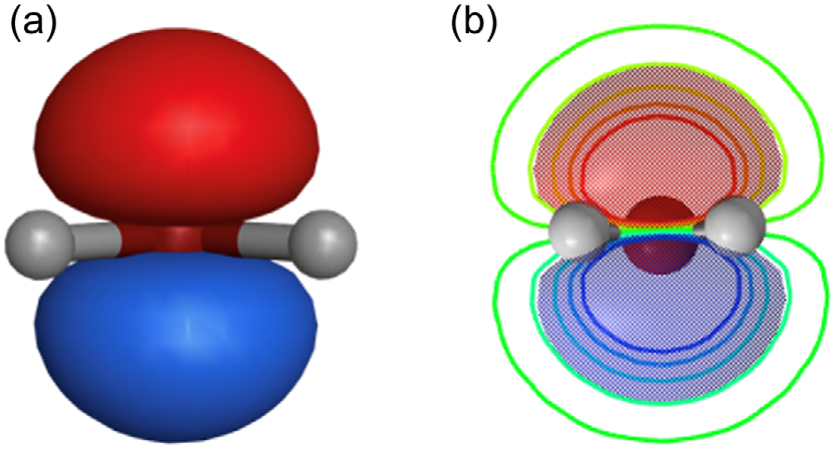
Representation of water HOMO using (a) arbitrarily chosen isosurface of water HOMO and (b) ad hoc plane slicing through HOMO orbital to display isocontour lines.

**Figure 5:**
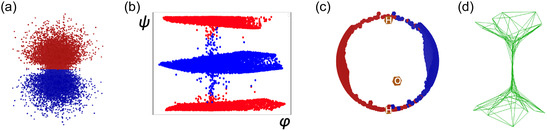
SGTM input and output files: (a) Sample of electron densities of water HOMO used as frame set. (b) The corresponding SGTM 2D map projection. The *x*‐axis is azimuthal angle (*φ*), and *y*‐axis is polar angle (*ψ*); (c) The 3D SGTM unit sphere projection; the oxygen and hydrogen atoms are also projected on this map for convenience. (d) Mesh representation of the SGTM manifold.

**Figure 6:**
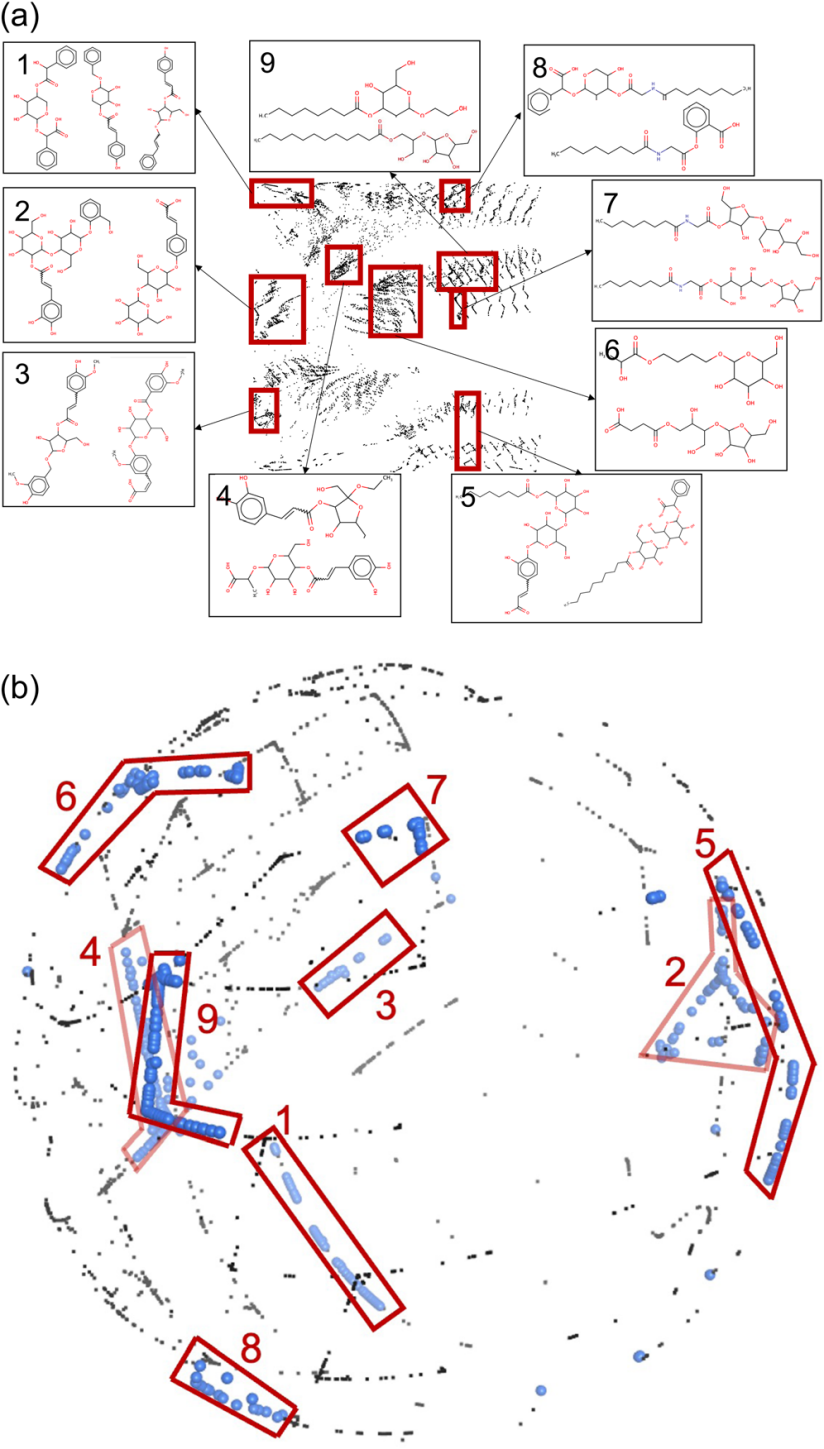
(a) The GTM and (b) the SGTM unit sphere projection of the tangible chemical library. Black dots are molecules. Areas without any molecule projections are white. Nine highlighted red boxes locate clusters of compounds with shared structural characteristics (see main text). Representative compounds of these clusters are exemplified on the map. In (b) for better visualization, the compounds inside clusters are represented as blue dots.

**Figure 7:**
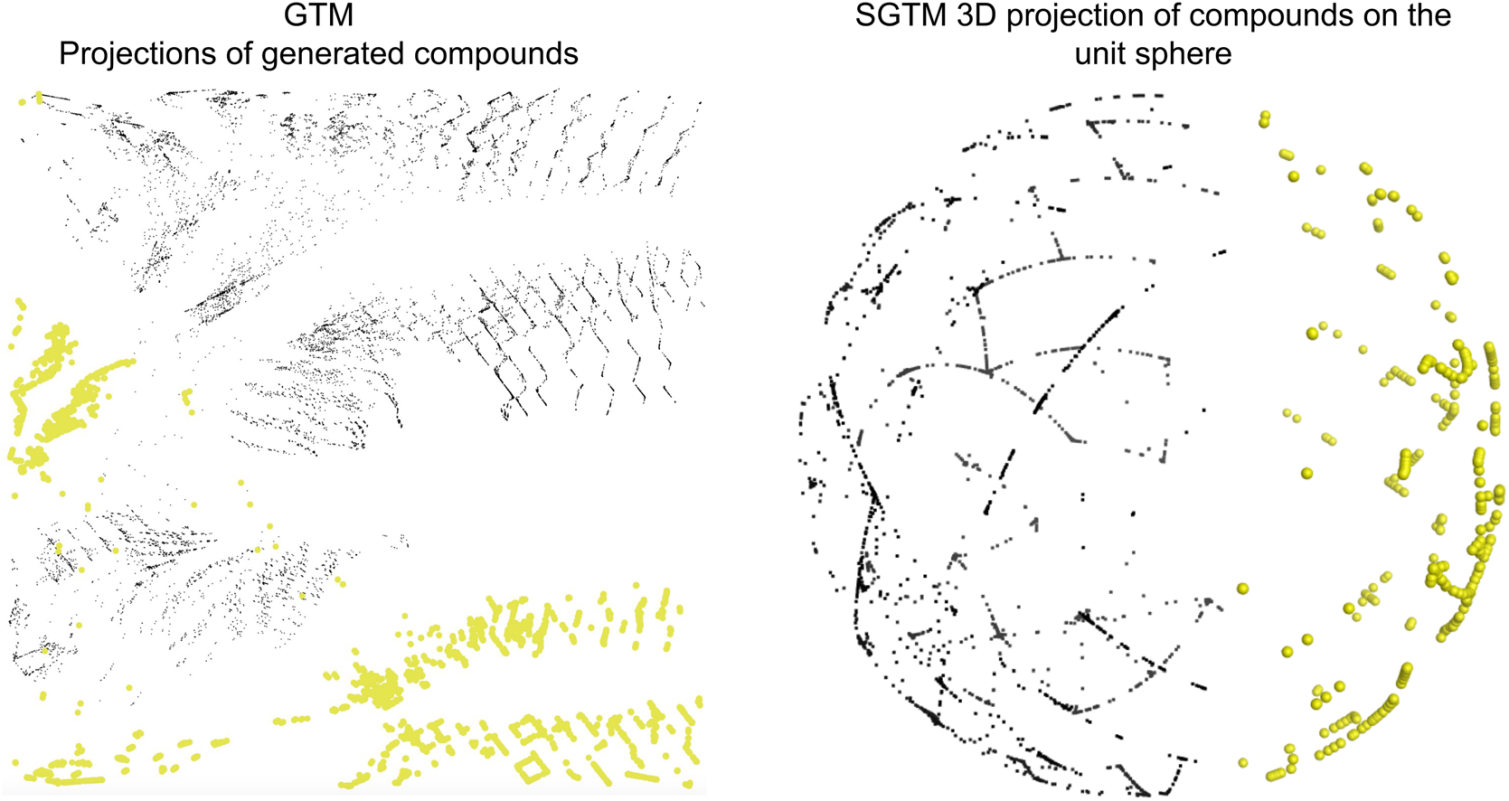
Comparative projections of the enumerated dataset onto the manifold using GTM and SGTM. On the left, the molecules containing disaccharides in yellow and green are split between the “south” and the “west” of the map. On the right, the dataset SGTM projection, where the molecular structures containing disaccharides are concentrated on the right half of the unit sphere map. The black dots represent the rest of the CosMoPoly library. The figures were prepared using MOE (version 2024.06).

Each model is constructed following the procedure outlined in the methods section. The parameter values for the number and width of the RBFs, along with the regularization coefficient, were identified by choosing the configuration of maximal likelihood. Figure 2 illustrates in the benzene electron density example, the evolution of the maximized likelihood at a given parameter set. These curves are analogous to those obtained in the other examples, including the electron densities and molecular orbitals of water and benzene shown in the SI (Figures 3, 4, 5, 6, 7).

The curve depicted in Figure 2a demonstrates how the *m* parameter affects the shape of the manifolds on the frame set. For the manifold fitted to the benzene electron density frame set at *m *= 20, the manifold has a star shape with an approximate C_6_ symmetry group. In this case, the manifold cannot fully capture the electronic distribution of both carbon and hydrogen. On the other hand, when *m *= 120, the manifold becomes more tightly aligned with the atoms of the molecule, except for a few nodes that extend outward and point toward regions that appeared slightly denser in the frame set but do not differ from other equivalents. As expected, a larger *m* value increases the model's capacity to fit the data, allowing it to capture more complex and detailed structures within the dataset.

Figure 2b illustrates how changing the regularization coefficient *l* affects the visualization. When the regularization parameter exceeds 1, the visualization becomes less detailed and captures fewer molecular features. Conversely, a very small value of *l* results in a more detailed visualization that may reflect finer distinctions, although some details may not generalize well across the dataset.

Figure 2c shows how the RBF width w affects the visualizations. A large value of *w* results in a smoother, more generalized visualization, while a small value leads to a more detailed visualization, potentially capturing subtle differences in the data. The optimal parameter value for *w* appears to be in the range of 0.2–0.4, balancing detail and generalization.

The scanning of the parameters of the manifold depends also on the complexity of the target distribution. For instance, the HOMO of water (Figure 3c) has a nodal plane where the wave function is null. Yet, the SGTM probability density cannot be canceled. Therefore, to minimize the error, it must have enough degrees of freedom to create a pinch going through this region of space. Thus, the value of the parameter *m* and *l* needs to be large enough. In this example, *m ≈ *80 (Figure 3a). At large *l* values (>100), the manifold takes a more simple and regular shape (Figure 3b).

The optimal values of the SGTM parameters, following a sequential procedure, are summarized in (Table S1, Supporting Information).

The number of RBF centers can be chosen in the order of magnitude 100 for molecules of molecular weights analogous to the examples presented herein. However, it is expected that this number grows linearly with the size of the molecule. The optimal value for the regularization coefficient is typically chosen as 1. But it can be increased to better capture the surroundings of singularities in the orbitals—nodal points where they cancel. The RBF width is better to be set by default; otherwise, the value 0.2 can be used as an initial guess.

#### 3.1.2 SGTM Data Visualization

Visualizing molecular orbitals and electron density is fundamental in computational chemistry and physics for understanding molecular interactions, bonding, and physical properties. Common techniques such as DFT use software like Gaussian and Pymol to transform computational data into 3D models [21]. However, these methods often rely on arbitrary choices, such as the selection of isosurfaces to represent wavefunctions or electron density (Figure 4a). The standard isovalue of 0.002 electrons au^−3^ is widely accepted as accurately portraying the size and shape of a molecule (for electron density) and the localization of wavefunctions [22]. While this value is commonly used, it remains somewhat arbitrary, and selecting the appropriate isovalue is critical for accurately interpreting molecular electron density.

An alternative method involves plotting isovalue curves on a section plane, but this also requires choosing an ad hoc plane to slice the orbital or density. This selection affects how the isocontour lines appear and can impact the interpretation of the data (Figure 4b). In contrast, SGTM does not necessitate such a choice; the manifold naturally finds its optimal alignment with the target density. The SGTM latent surface has an interpretation as the location in space with the maximal expectation to find an electron.

For illustration, the frame dataset is the 3D coordinates of electrons sampled from the water HOMO orbital (Figure 5a). The dataset is projected on the SGTM manifold, which is mapped back to a unit sphere; the unit sphere is flattened using an equirectangular projection. The resulting 2D map is independent of the molecule's orientation in space, yet it remains sensitive to its conformation (Figure 5b). On the corresponding unit sphere (Figure 5c), the division between the two lobes of opposite parity in the HOMO is clearly visible, and the symmetry of the compound emerges naturally, albeit approximately. Generally, SGTM's unit sphere views highlight topological features of the modelled densities and orbitals.

In the present case, the spherical manifold is embedded in a 3D data space. To visualize the manifold a mesh representation is used, where nodes within the mesh correspond to the manifold's nodes. A connection is established between two nodes if they are adjacent on the unit sphere that maps the manifold (Figure 5d).

The use of the unit sphere to represent the projections is illustrated in Figure 5b,c. The two lobes of the HOMO are expected to generate two distinct populations of electrons. In Figure 5b, the periodicity of the *x*‐ and *y*‐axis is not obvious, so it seems that there are three populations of electrons. The visualization on the unit sphere (Figure 5c) can be less misleading.

Additionally, this approach is an example of using machine learning algorithm to transform a quantum chemistry solution into another mathematical expression.

$$DFT\;\;\overset{\text{GTM}}{\to }\;\;\text{Normal}\left(\phi ,\beta \right)$$

In the present case, this method builds a normal distribution centered on a manifold *ϕ* and width *β*, from DFT solutions. As future work, one could formulate the SGTM formalism as a basis function set, saving computational power for sampling and fitting a distribution that obeys known mathematical formulations. The computational cost is actually paid largely in the generation of the electrons position datasets. The SGTM, on the other hand, needs about one minute to fit 20,000 data points on a standard laptop computer.

We conducted an additional experiment using the HOMO of water frame set. Starting from the original frame set of 20,000 points, we progressively reduced the size of the dataset by a factor of two, testing the SGTM on 10,000, 5,000, 2,500, 1,250, 620, and 310 data points. The resulting SGTMs remained visually consistent across all levels of dataset reduction, demonstrating that the method converges with significantly fewer training points. These results are presented in (Figure S13, Supporting Information).

### 3.2 Analysis of Tangible Chemical Space

This explicitly enumerated dataset has been analyzed using both the GTM and SGTM techniques.

#### 3.2.1 Dataset Generation

The CosMoPoly dataset comprises 25,289 molecules. This molecular dataset was generated by systematically enumerating BBs through predefined reaction, resulting in many clusters of structural analogues.

Figure 6a illustrates a GTM and 6.b a SGTM projection onto a unit sphere of molecular structures derived from the enumeration of BBs. The black dots on the map are the compounds. The white areas are places where no molecules from the dataset are located; it identifies chemical space regions not covered by the tangible chemical space. Through visual inspection of the density landscape map (see Figure S12, Supporting Information), we could characterize nine large clusters of compounds sharing structural features, as described in the following:

1. Compounds with carbohydrates and two benzene rings attached at opposite sides of carbohydrates—208 compounds.
2. Disaccharides linked to a benzene ring—1590 compounds.
3. Carbohydrates attached to a benzene ring, with a hydroxyl hydrogen atom substituted by a methyl group—385 compounds.
4. Carbohydrates linked to alcohols via the hydroxyl group at the C1 position and to acids through other hydroxyl groups—643 compounds.
5. Disaccharides linked to an acid group, a phenolic ring, and an alkyl chain—1050 compounds.
6. Carbohydrates attached to acids through alcohol groups—1552 compounds.
7. Compounds similar to those in 5 but with additional nitrogen‐containing groups—228 compounds.
8. Phenolic compounds with nitrogen and alkyl chains.
9. Carbohydrates linked to both alcohol and acid groups, with the addition of alkyl chains—1397 compounds.

#### 3.2.2 Difference between GTM and SGTM Visualization of Density Landscape

The likelihood is used here to compare the two maps, with GTM scoring −63.35 and SGTM achieving −57.22—SGTM fitting the dataset a bit better. The projections reveal clear segregation of disaccharides containing compounds, i.e., those with pyranose–furanose or pyranose–pyranose motif. The GTM (Figure 7, left) shows these compounds, in yellow distributed on homogeneous regions on the “south” and “west” of the map. We observed that the “west” region appears isolated. We hypothesize that it is an artifact. This effect could be explained by the bending of the manifold, using disjoint parts of it to fit the chemical space where disaccharide‐containing compounds are sitting. The SGTM, in contrast, presents these compounds on one half of the spherical map. This distribution on the other hand is more packed: more compounds are concentrated in tiny regions of this unit sphere map (Figure 7).

**Figure 8:**
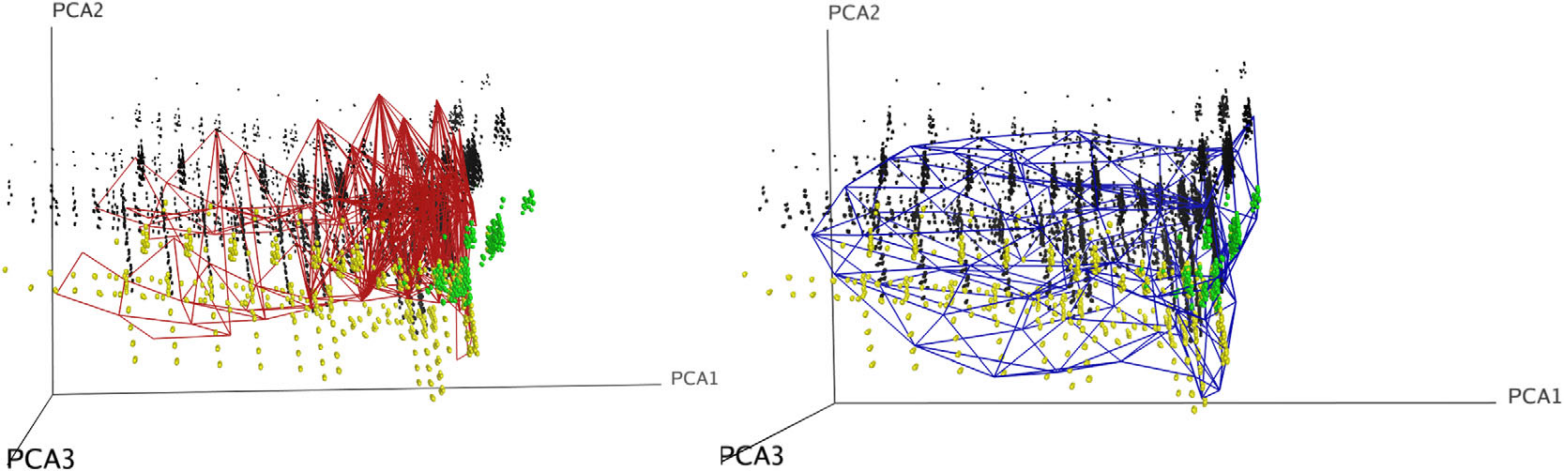
Highlighted in yellow and green are the disaccharide‐containing compounds; black data points are other compounds from the CosMoPoly dataset. The green data points correspond to the yellow data points cloud located on the “west” of the GTM map in Figure 7. The GTM manifold (red mesh on the left) does not fit the green region, whereas the SGTM manifold (blue mesh on the right) does. The black dots represent the rest of the CosMoPoly library.

This interpretation is illustrated in Figure 8 using projections on the first three principal components of the datasets. The GTM and SGTM manifolds are overlapped, as mesh, to the data points representing the molecules. The yellow data points correspond to those compounds located at the “south” of the map and the green data points are identifying those on the “west.” The GTM manifold does not appear to go “through” the green data points cloud; instead, it uses a portion of the manifold to locate them on the map. On the other hand, as the SGTM has no borders, it preserves more easily the integrity of such clusters. As a result, the disaccharide‐containing compounds are populating the same half of the unit sphere map.

## 4 Conclusions

This study introduces the Spherical GTM (SGTM) method. This novel approach represents a significant addition to the GTM method by usemploying a spherical manifold that better captures the nature of data possessing the topology of a sphere.

To achieve this, the GTM has been modified in three key aspects: (1) using spherical coordinates to locate RBF centers and nodes, (2) using a quasiregular grid to generate coordinates of RBF centers and nodes, and (3) the initialization of the manifold.

The SGTM algorithm is benchmarked on a mapping of electronic densities and molecular orbitals. It produced an original visualization of the electron densities and molecular orbitals of the selected molecules. The method's free parameters, including the number of RBF centers, regularization coefficient, and RBF width, have been explored to illustrate their effect on the fitting of the data. Systematic scanning of parameters’ values showed that for the medium sized dataset: (1) a number of RBF centeres, *m*, around 100 provides sufficient flexibility to capture detailed patterns in the datasets without overfitting; (2) the regularization coefficient, *l*, which controls expressivity of the manifold, can be initially set to 1, but higher values (typically 10–30) are advantageous when the dataset includes nodal planes or other singularities; and (3) a RBF width, *w*, between 0.2 and 0.4 offers an effective balance between local detail and overall smoothness. These values are good starting points for new systems of comparable size; finer tuning can then be guided by visual inspection of the manifold and unit sphere projection.

Then, the GTM and SGTM analysis of tangible chemical space have been compared. It shows that SGTM brings on the same dataset another perspective that can be more relevant or can complement the GTM. Since the spherical manifold has no borders, SGTM avoids the edge folding artifacts sometimes observed in GTM and preserves the integrity of large, contiguous clusters (e.g., the disaccharide subset of the CosMoPoly dataset, in this case).

Of course, it is difficult to “visualize” the topology of the high‐dimensional data before mapping—therefore, both GTM and SGTM should be used concurrently, in order to find the map maximizing the user's expectations (minimal loss, or, alternatively, landscapes of maximal predictivity).

The regularization parameter can be used for chemical space visualization, in order to generate a smoother map (see Figure S13, Supporting Information). As the map becomes more smooth, the projections are more evenly distributed over the manifold. On the other hand, a less regularized model tends to tighten up the projections of the molecules on the map.

The loss function in both GTM and SGTM is the same. It results from the maximization of the likelihood of the dataset using the same kind of probability distribution. One is centered on the bounded manifold; the other uses a surface topologically equivalent to a sphere. For this reason, we propose that the log likelihood obtained by both methods on the same datasets are quantitatively comparable.

Thus, SGTM can be particularly useful for data that are hypothesized to possess an spherical topology or data where imposing a spherical topology can bring another perspective, for instance, to detect if a cluster structures visually identified on a GTM is genuine or results from a boundary artifact.

Future work includes exploring the potential of SGTM to visualize the phase space of electrons, capturing on SGTM dynamic properties of molecular systems and formulating GTM for other topologies.

## Conflicts of Interest

The authors declare no conflict of interest.

## Supporting information

Supplementary Material

## Data Availability

The CosMoPoly dataset that supports this study is freely available on Recherche Data Gouv at https://doi.org/10.57745/RAHSKA. The SGTM software and source code are available upon request to the corresponding author.
