# Supplementary material for: Spherical GTM: A New Proposition for Visualization of Chemical Data
Source: Mol Inform. 2025 Jun 16;44(5-6):e2500045. doi: 10.1002/minf.202500045 (PMC12186103; doi:10.1002/minf.202500045)
Supplement: Supplementary file 1 — Supplementary Material [file MINF-44-e2500045-s001.pdf]

Support Information

Spherical GTM: a new proposition for visualization of chemical data

Farah Asgarkhanova, Gilles Marcou, Mikhail Volkov, Murielle Muzard, Richard Plantier-Royon, Rémond Caroline , Dragos Horvat, Alexandre Varnek

Table of content

Ab initio electronic structure calculations	3
SGTM parameters exploration	4
Evolution of the frame set likelihood for electron density, HOMO, and LUMO for water and benzene	7
BBs and reaction rules used for systematic enumeration to create tangible data space	12
Parameter selection for GTM for analysing tangible chemical space	15
CosMoPoly density landscape	16
CosMoPoly projection on a unit sphere	17
Application of SGTM on Frame Sets of Varying Sizes	18
Application of SGTM on Tox21 database	20

Ab initio electronic structure calculations

The ORCA toolkit (v. 4.2.1) was used to generate the electronic densities, Highest Occupied Molecular Orbital (HOMO), and Lowest Unoccupied Molecular Orbital (LUMO) of molecules to illustrate the concept.

The following procedure was applied (Figure 1):

1. Quantum Calculations:
    - Density Functional Theory (DFT) with a B3LYP hybrid functional.
    - The Karlsruhe basis set DEF2-SVP.
    - Optimization of the molecular geometry at this level of theory.
2. Procedure:
    1. Estimation of the electronic density $\phi(x_k)$ or an orbital $\Psi_i(x_k)$ at the nodes x_k of a 3D regular cubic grid (40x40x40 nodes).
    2. Computing the probability $P(x_k)$ of observing an electron in a cubic volume centred on x_k.
        i. For electronic distribution: $P(x_k) \propto \phi(x_k)$.
        ii. For the ith orbital: $P(x_k) \propto |\Psi_i(x_k)|^2$.
3. Building a partition function $PF(x_k)$ by recursion:
    i. $PF(x_k) = PF(x_{k-1}) + P(x_k)$, with $PF(x_0)$ = 0.
4. Choosing a random number r within the interval [0,1], which uniquely identifies a grid node x_k as $PF(x\_(k-1))$≤r<$PF(x\_k)$.
5. Sampling one coordinate set from a uniform distribution inside the cube centred on x_k.
6. If the molecule is symmetric, repeat the sampling process from step 4 until half of the desired number of data points is reached. Generating a symmetrical half of the dataset.
7. If the molecule is not symmetric, repeat the sampling process from step 4 until the desired number of data points is achieved.

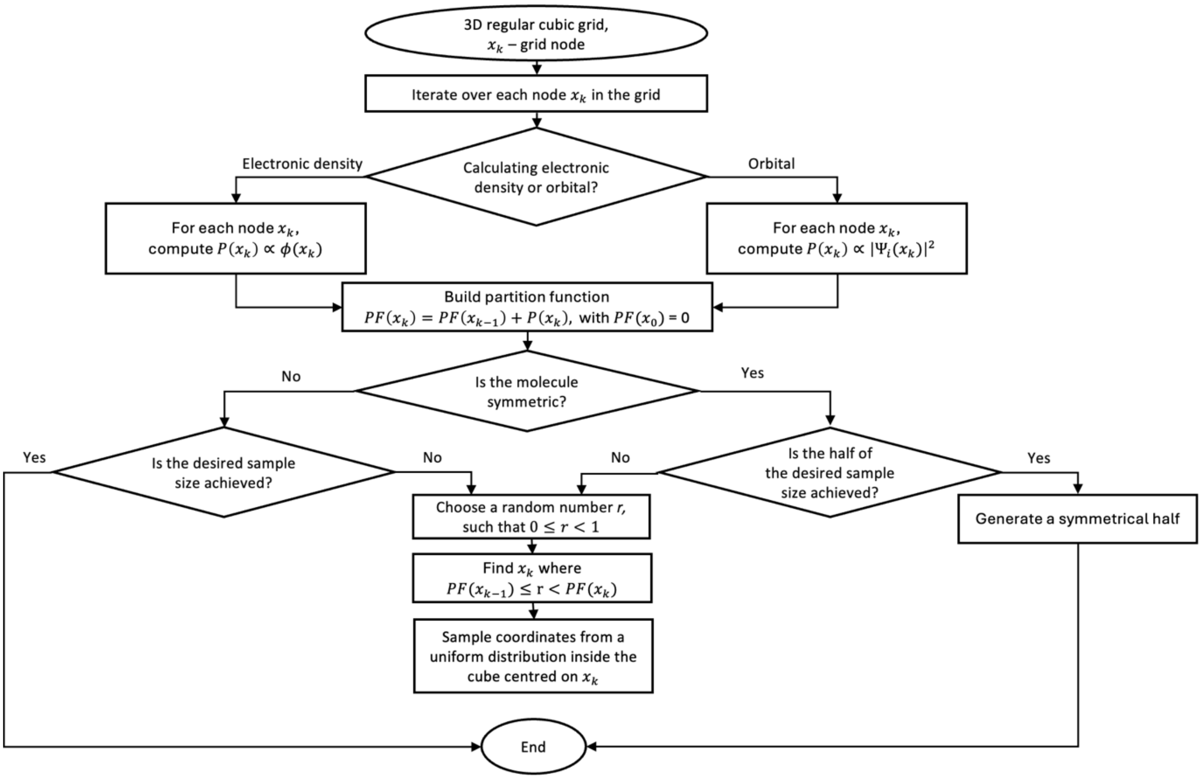

Figure 1. Quantum Mechanics on a 3D Cubic Grid: A Step-by-Step Process Flow: 1. Iterating over each node in a 40x40x40 grid. 2. Estimating electronic density or orbital probability. 3. Computing probabilities for electron presence in cubic volumes. 4. Recursively building the partition function for probabilities. 5. Using random sampling within [0,1] to select grid nodes. 6. Sampling coordinate sets inside the cubes. 7. Repeating until the desired sample size is achieved.

SGTM parameters exploration

The process for fitting parameters was methodically applied (Figure 2):

1. Creation of Frame and Validation Sets (see paragraph "*ab initio* electronic structure calculations")
   a. Generate a frame set with a sample size equal to 20000. The data was centred.
   b. Generate independently a validation set for evaluation purposes.
2. Tuning the Number of Radial Basis Functions (RBF) Parameter, *m*
   a. Launch the SGTM, varying the number *m* of RBF from 20 to 125 in increments of 20. All other parameters are set to default value.
3. Model evaluation and parameter exploration
   a. Record the likelihood scores for each model.
   b. Identify the parameter value that yields the highest likelihood score.
4. Tuning the regularization coefficient parameter, *l*
   a. Use the selected *m* value previously computed in step (3.c). Iteratively launch the SGTM, adjusting the regularization coefficient *l* from 0.3 to 1 in increments of 0.3, then from 2 to 10 in increments of 2, and then from 10 to 100 in increments of 20.
   b. Repeat the evaluation and parameter exploring process (step 3) to identify the most suitable *l* value.
5. Tuning the RBF width parameter, *w*
   a. Use the previously selected values of *m* and *l* (3.c and 4.a). Iterate the SGTM's RBF width parameter from 0.1 to 1 in 0.1 increments.
   b. Repeat the evaluation and parameter exploring process (step 3) to identify the most suitable *w* value.

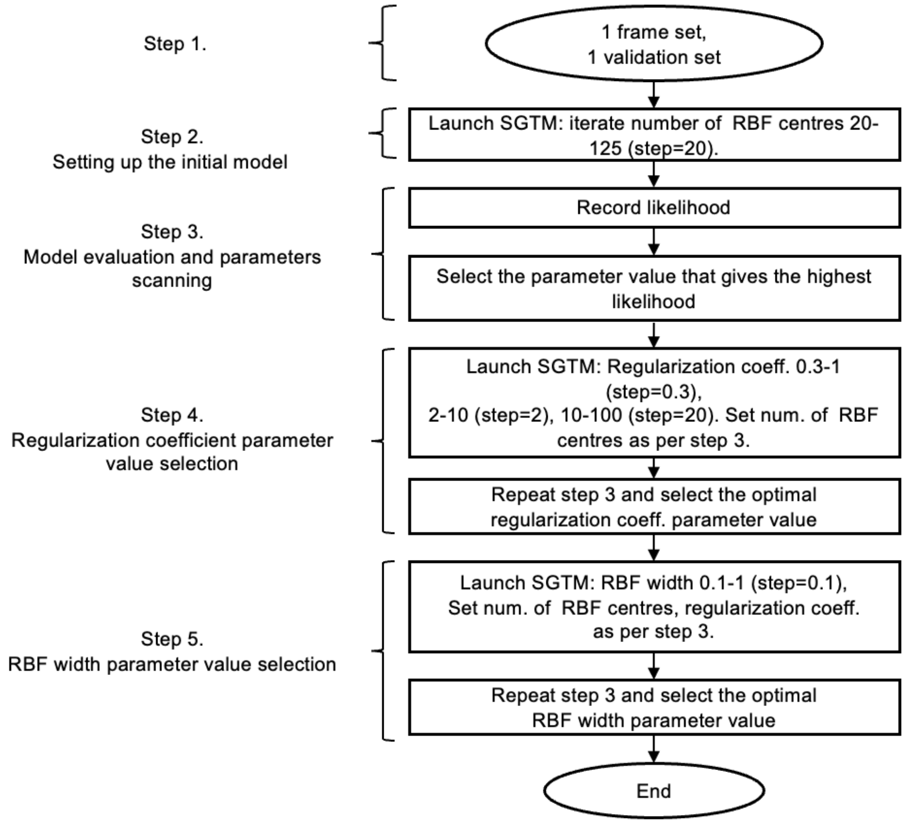

Figure 2. Parameter exploring process for SGTM. This figure outlines a methodical approach for scanning SGTM parameters across 5 steps: (1) Generation of 1 frame set to build the model, (2) Initial configuration of SGTM with varying numbers of RBF centres to determine their impact on performance, (3) Performance assessment and parameter selection based on likelihood scores, (4) Selecting the optimal regularization coefficient value, (5) Selection of the optimal RBF width parameter value.

Table 1. The optimal values and the likelihood of the final SGTM models. The values' parameters are determined through a sequential procedure. The number of RBF centres is typically around 100 for molecules with molecular weights similar to those in our examples. The regularization coefficient is optimally set at 0.3. However, very large values can be preferred to obtain more simple manifold shapes and stable models (less sensitive to parameter values). The RBF width is recommended to be maintained at the default value.

	Number of RBFs, m	Regularization coefficient, l	RBF width, w	Likelihood
Benzene electron density	100	0.3	0.2	-5.45
Benzene HOMO	100	1	0.13	-5.47
Benzene LUMO	100	2	0.13	-5.61
Water electron density	120	0.6	0.13	-3.63
Water HOMO	80	100	0.2	-3.49
Water LUMO	120	2	0.13	-4.67

Figure 3. (a, b, c) Evolution of the frame set likelihood for water electron density as a function of SGTM free parameter variations. (d) Frame set.

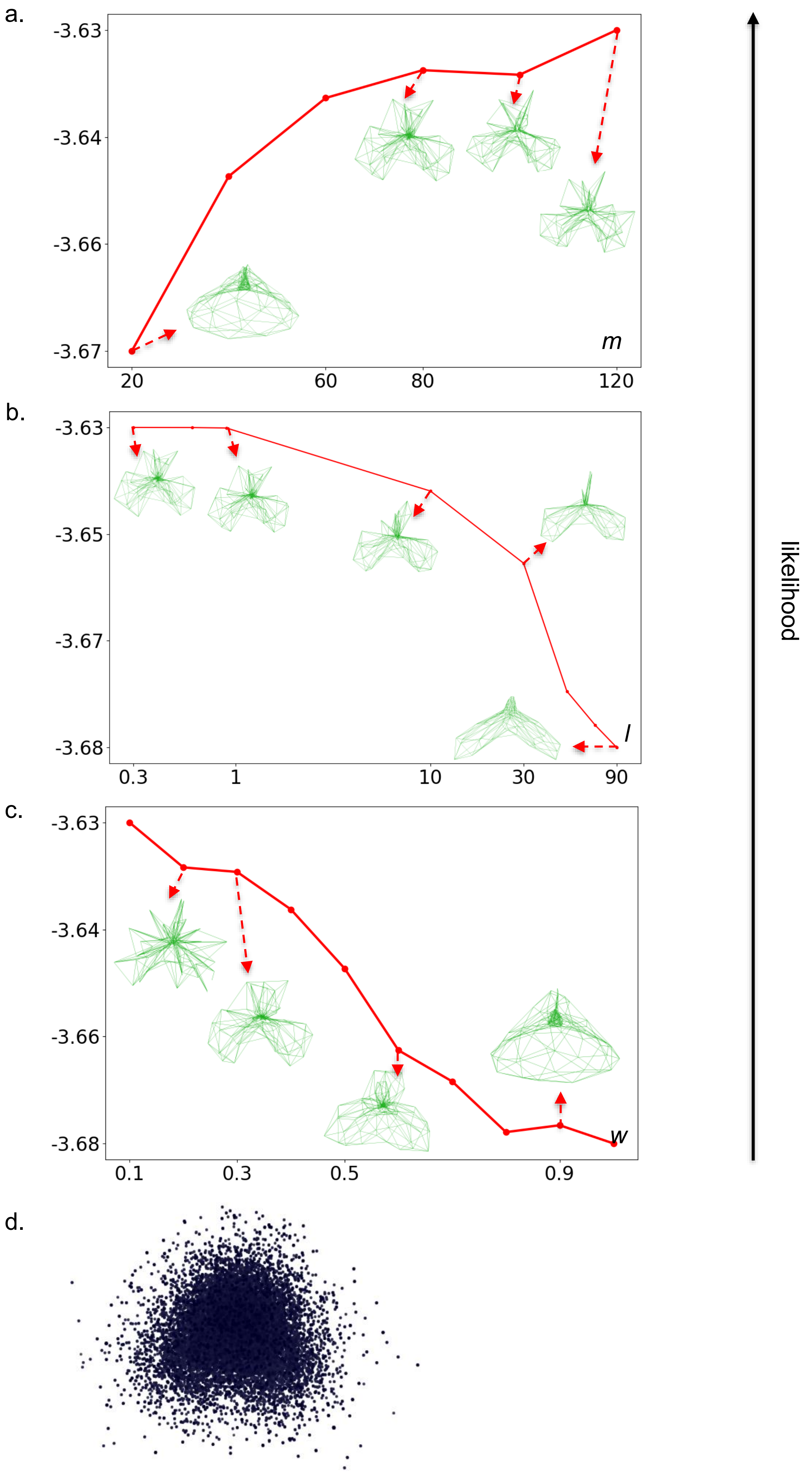

Figure 4. (a, b, c) Evolution of the frame set likelihood for water LUMO as a function of SGTM free parameter variations. (d) Frame set.

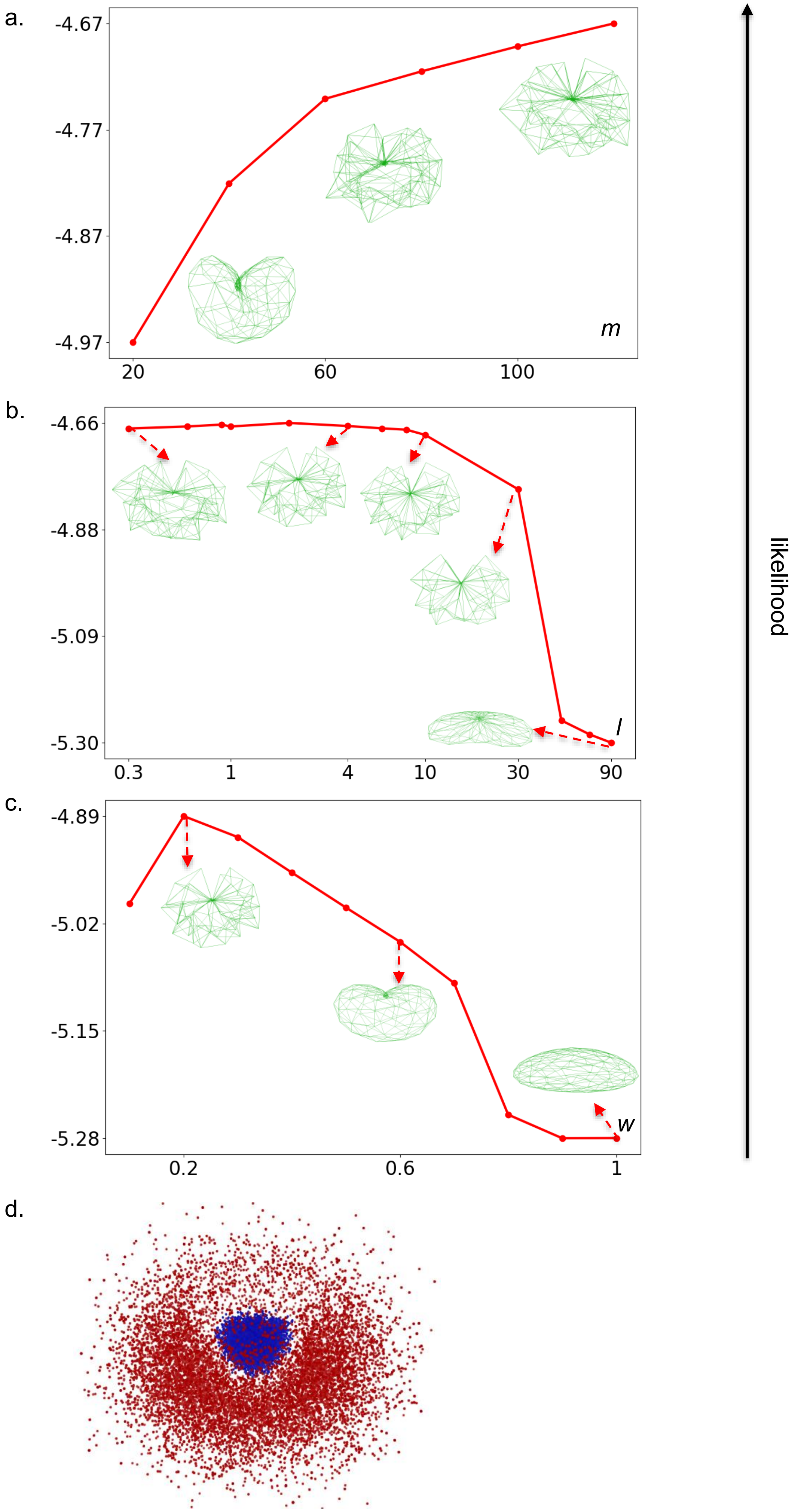

Figure 5. (a) Evolution of the frame set likelihood for water HOMO as a function of SGTM free parameter variations. (d) Frame set.

a.

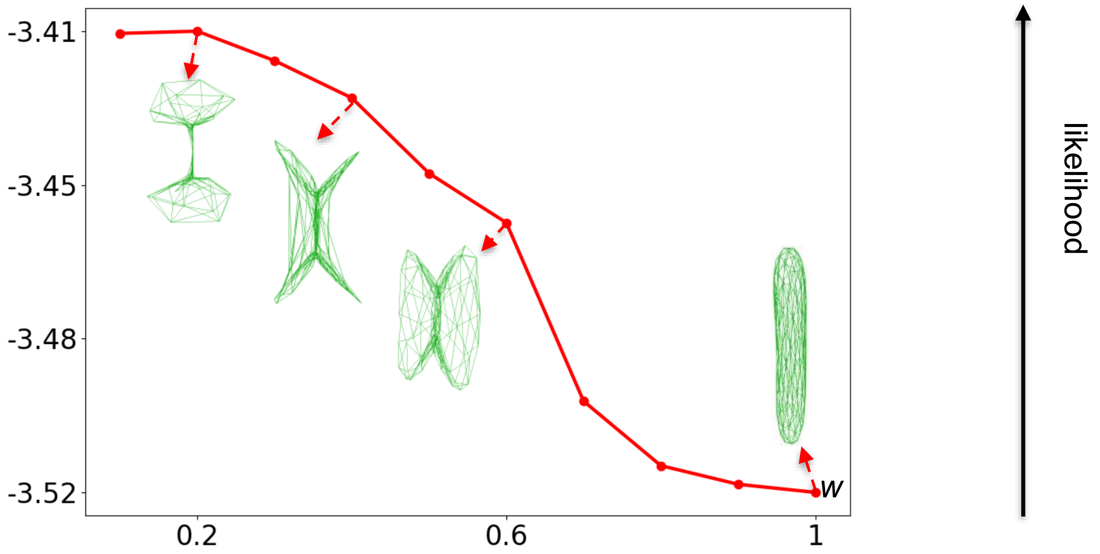

b.

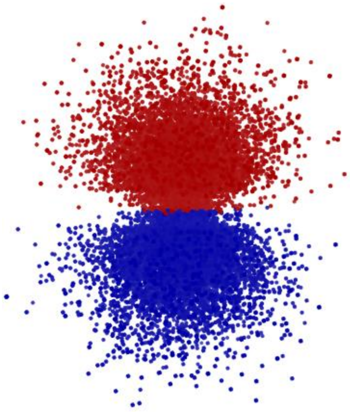

Figure 6. (a, b, c) Evolution of the frame set likelihood for benzene HOMO as a function of SGTM free parameter variations. (d) Frame set.

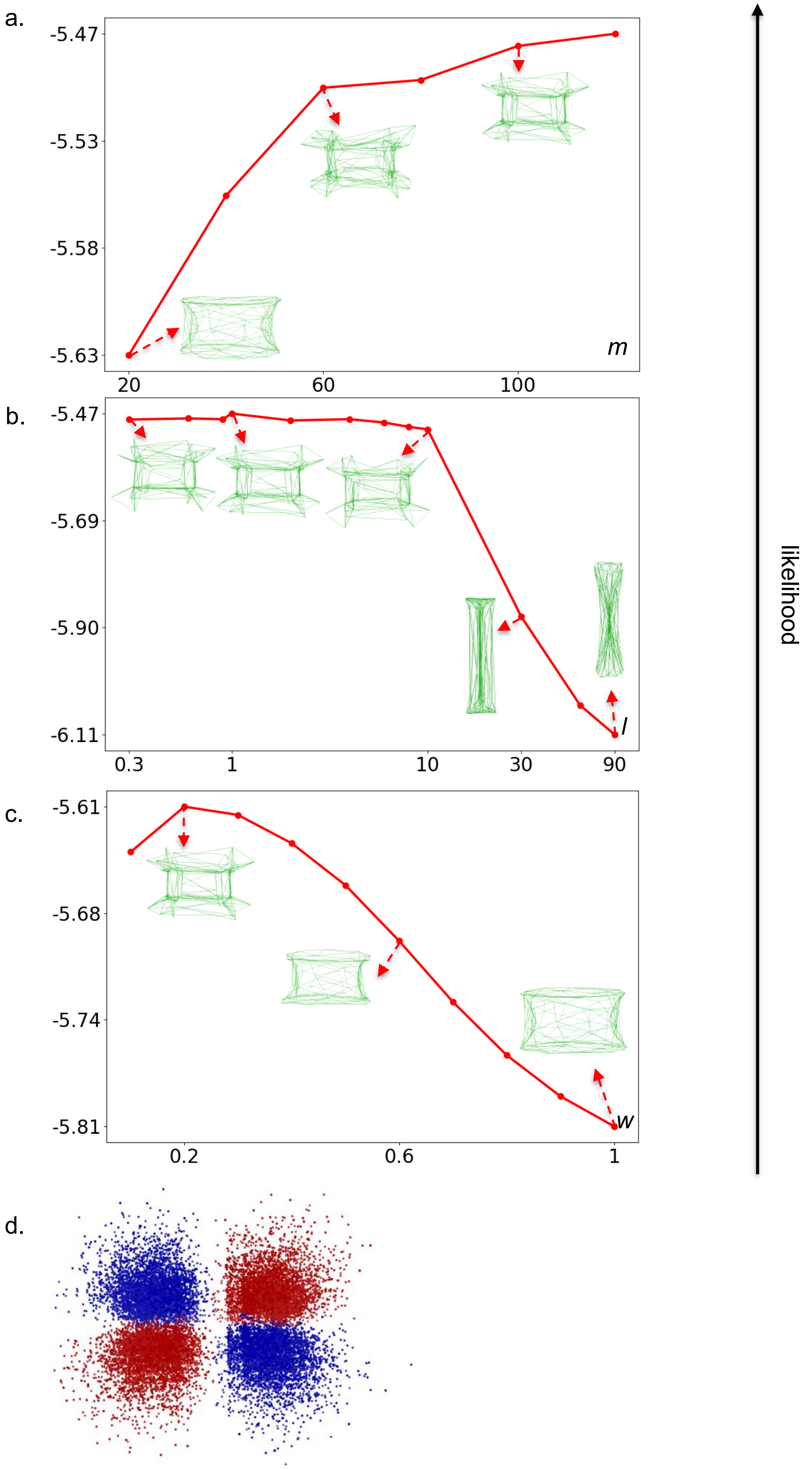

Figure 7. (a, b, c) Evolution of the frame set likelihood for benzene LUMO as a function of SGTM free parameter variations. (d) Frame set.

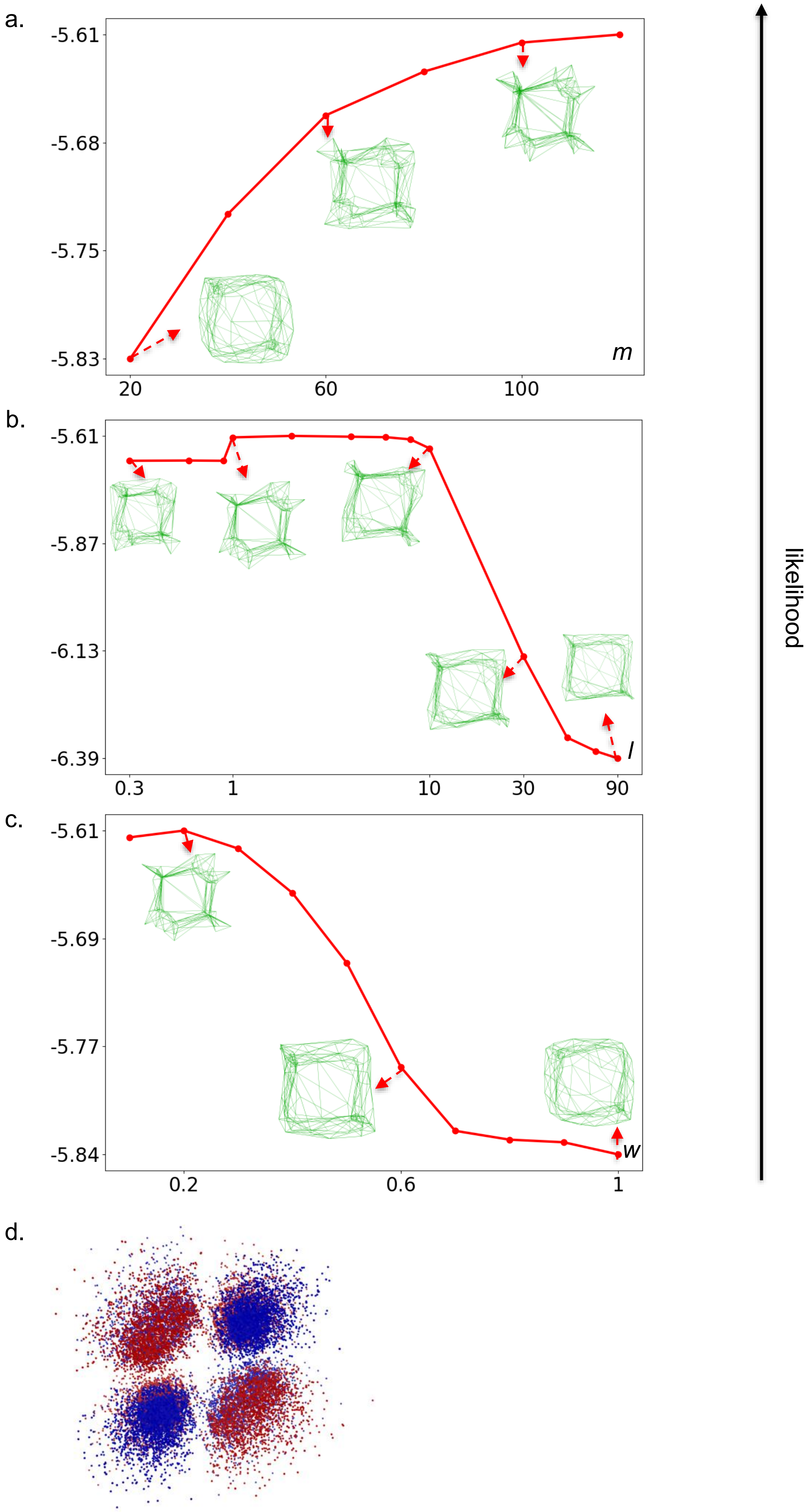

BBs used for systematic enumeration to create tangible data space.

1.D-Glucopyranose
2.D-Xylopyranose
3.L-Arabinofuranose
4.D-Mannopyranose
5.D-Galactopyranose
6.D-Fructofuranose
7.Sucrose
8.Maltose
9.Caprylic acid
10.Capric acid
11.Lauric acid
12.Myristic acid
13.Stearic acid
14.Lactic acid
15.Succinic acid
16.Salicylic acid
17.Glycolic acid
18.Malic acid
19.Mandelic acid
20.Azelaic acid
21.Methanol
22.Ethanol
23.Butanol
24.Propanol
25.Pentanol
26.Hexanol
27.Octanol
28.Benzyl alcohol
29.Cinnamyl alcohol
30.Vanillyl alcohol
31.Salicyl alcohol
32.1,4-Butanediol
33.1,6-Hexanediol
34.Octane-1,2-diol
35.Ethylene glycol (1,2-ethanediol)
36.Sorbitol
37.Xylitol
38.Resveratrol
39.Glycerol
40.Erythritol
41.Ferulic acid
42.Caffeic acid
43.p-Coumaric acid
44.Sinapic acid
45.Vanillic acid
46.Dihydroferulic acid
47.Capryloyl glycine
48.Vanillin
49.N-Palmitoylglycine

Reaction rules used for enumeration os BBs

A Building Block (BB) is a small molecule that includes at least one functional group. These 48 BBs include carbohydrates, alcohols, and acids. These BBs and 2 predefined reaction rules were chosen because they are readily accessible to synthetic chemists in the laboratory. To systematically enumerate BBs, synthons were created. A synthon is a molecular fragment which is created in the following steps using the predefined fragmentation reaction rules:

1. Removing the leaving group.
2. Adding the reaction centre label.

After synthons creation, all synthons are combined using the predefined reaction rules to form the final product. This approach enables the systematic enumeration of all compounds that can be synthesised following a planned synthetic route (Figure 8).

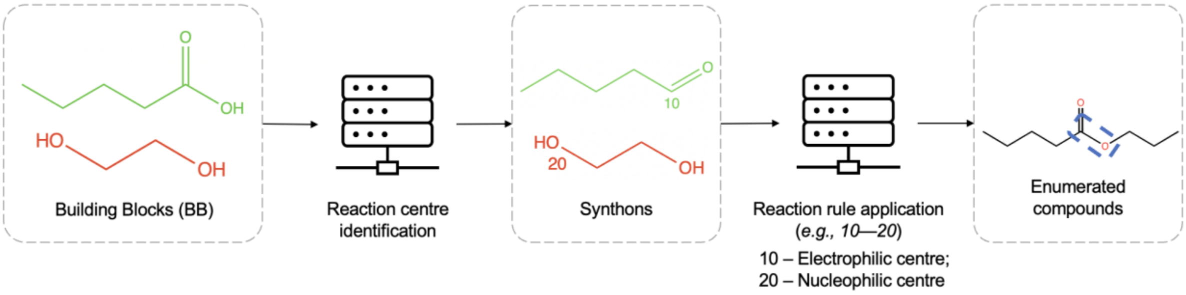

Figure 8. The process of enumerating the tangible chemical space from building blocks. Using the predefined fragmentation reaction rules, synthons are created from BBs by removing the leaving group and adding the reaction centre label. Subsequently, the synthons are combined using predefined reaction rules for enumeration. In this example, an acid with label 10 (electrophilic centre) and an alcohol with label 20 (nucleophilic centre) are coupled through the esterification reaction (10 and 20 coupling) to form an ester.

A detailed explanation of the two reaction rules used during the enumeration process is provided below:

1. Acetalization followed by esterification reaction rule (Figure 9).
    1. Synthons are created from carbohydrates and polyols/diols using predefined fragmentation reaction rules, where the reaction centre label 10 identifies electrophilic centres and label 20 identifies nucleophilic centres.
    2. These synthons are combined based on their reaction centre labels (10-20 coupling) to form acetals.
    3. Synthons are then created from the reaction products from step 3 (acetals) and alcohols.
    4. The newly created synthons are combined based on their reaction centre labels to form esters.
2. Esterification reaction rule (Figure 10).
    1. Synthons are created from simple alcohols, the hydroxyl group of carbohydrates (except the hydroxyl group at the C1 position), and acids using predefined fragmentation reaction rules. The reaction centre label 10 identifies electrophilic centres, and label 20 identifies nucleophilic centres.
    2. These synthons are combined based on their reaction centre labels (10-20 coupling) to form esters.

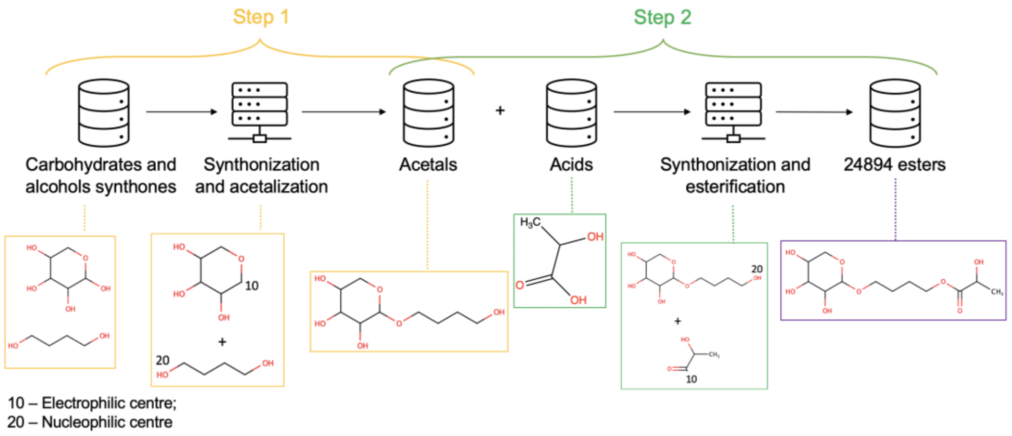

Figure 9. Systematic enumeration using acetalization and esterification reactions. Synthons are derived from carbohydrates and polyols/diols. The reaction centre label 10 identifies electrophilic centres, while label 20 represents nucleophilic centres. These synthons are then combined based on their reaction centres to form acetals. Subsequent synthons are generated from the acetal products from step 1 and alcohols, which are then combined to form esters. This process results in the total enumeration of 24894 esters derived from BBs.

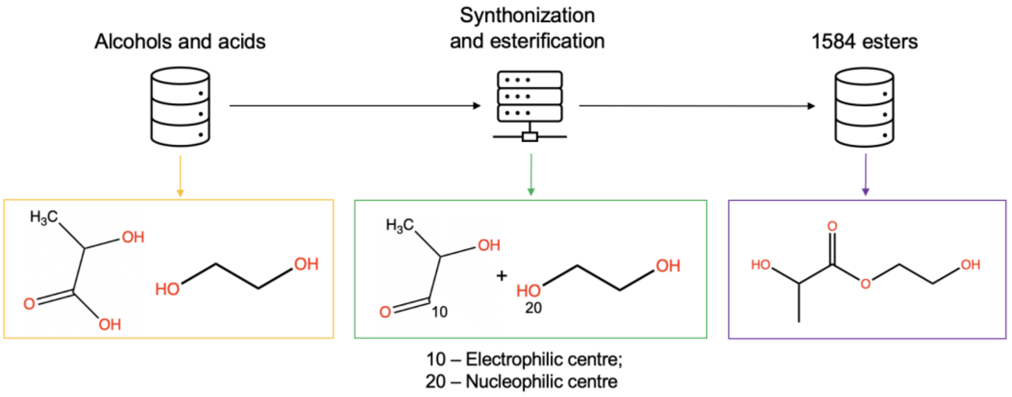

Figure 10. Representation of the ester generation process. Synthons are created from alcohols and acids using predefined fragmentation reaction rules, where the reaction centre label 10 identifies electrophilic centres, and label 20 identifies nucleophilic centres. The synthons are then combined to form esters according to the esterification reaction rule (10-20 coupling). This process results in the total enumeration of 1584 esters derived from BBs.

Parameter selection for GTM for analysing tangible chemical space

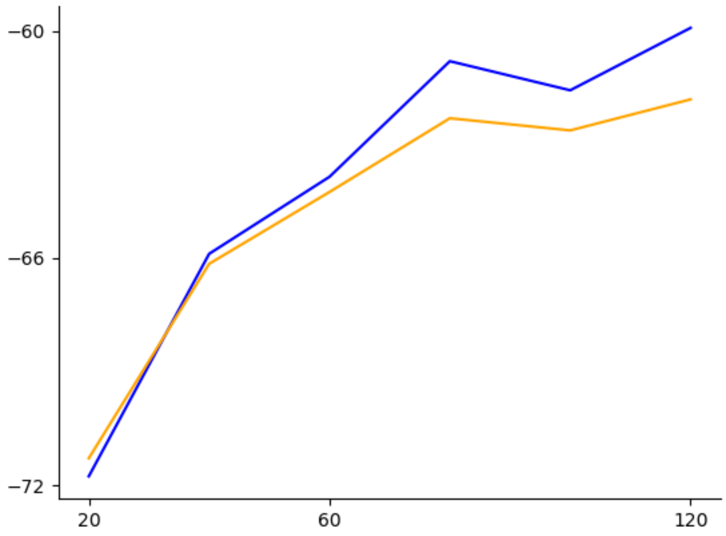

Figure 11. The number of RBF centres (m) parameter values (x-axis) vs the likelihood (y-axis) selected for GTM for analysing tangible chemical space. The frame set is in blue, and the validation set is in orange. At value 50, the model performs well on the frame dataset and generalises to the validation dataset.

Density landscape map of the CosMoPoly library

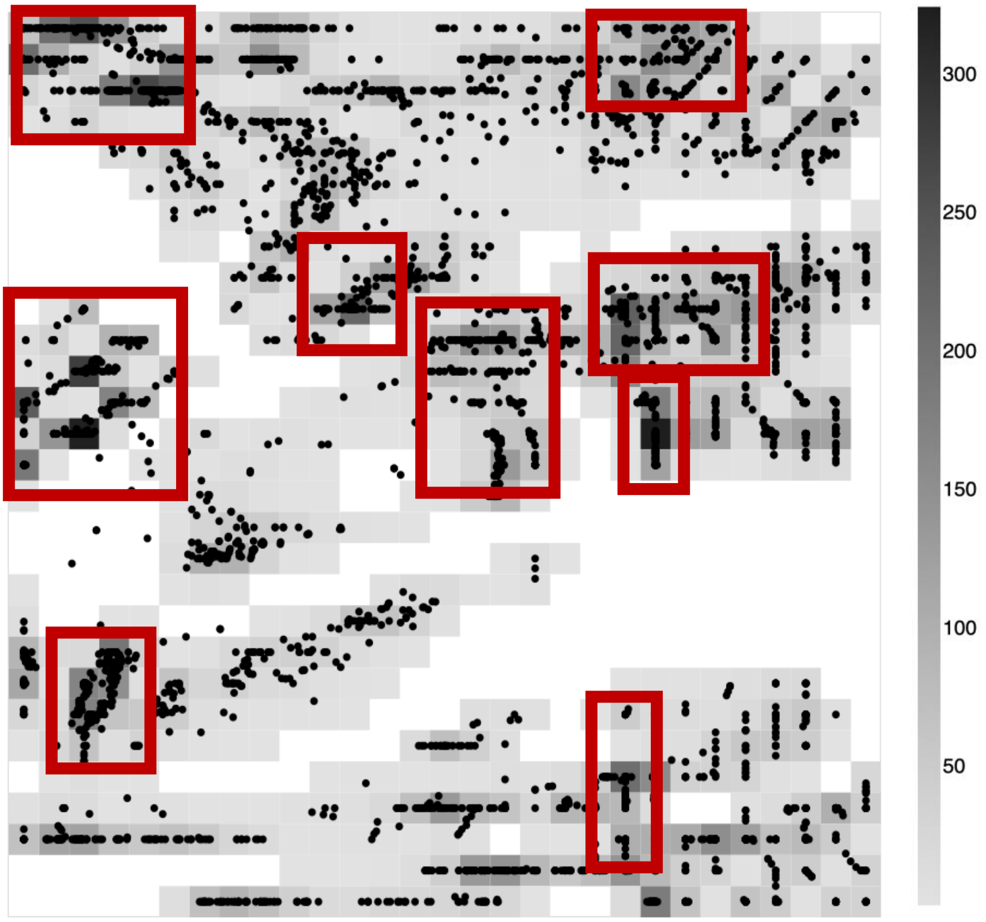

Figure 12. Density landscape map of the CosMoPoly library prepared using GTM. Black dots represent individual molecules, and darker colors indicate regions with higher molecular density.

CosMoPoly projection on a unit sphere

Figure 13. Increasing the regularization coefficient allows the lines on the SGTM projection to become more dispersed. The clusters are the same as in the main text (Figure 6.a). In the SGTM unit sphere projection, clusters 1 and 3 appear to be merged. This can be explained by the fact that, in the GTM map, these clusters are positioned near the corners, where they may be affected by border effects, and they also share very similar structural features.

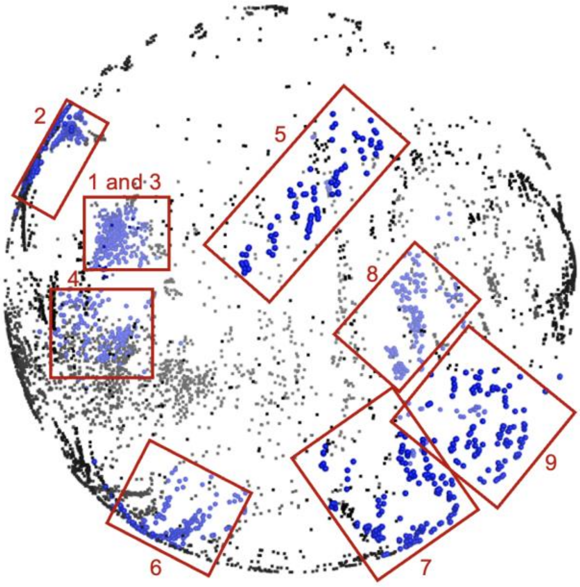

Application of SGTM on water HOMO frame Sets of Varying Sizes

Figure 14. SGTM input and output files: (a) Sample of electron densities of water HOMO used as frame set; (b) The corresponding SGTM 2D map projection. The x-axis is azimuthal angle (φ), and y-axis is polar angle (ψ); (c) The 3D SGTM unit sphere projection; (d) Mesh representation of the SGTM manifold.

1. Size of frame set = 10000 datapoints

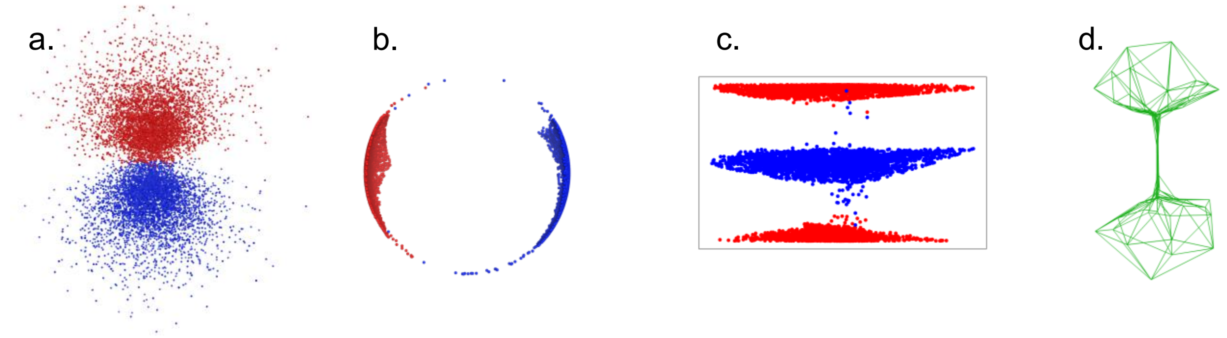

2. Size of frame set = 5000 datapoints

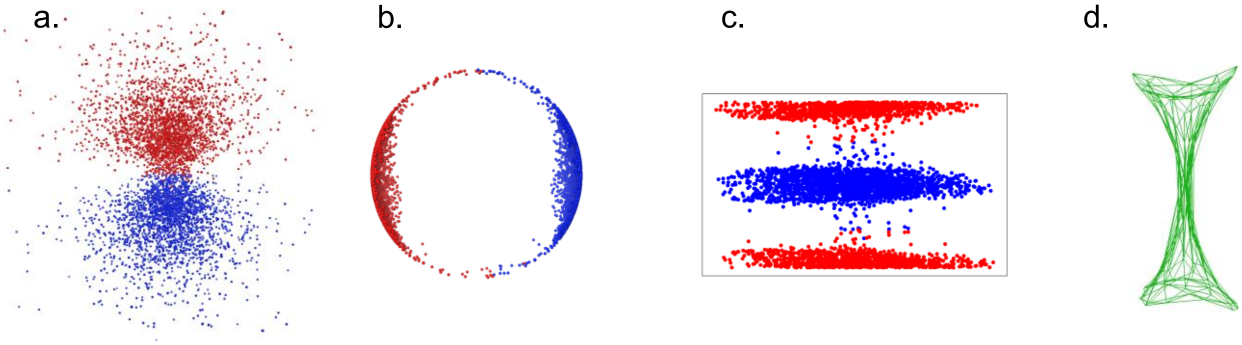

2. Size of frame set = 2500 datapoints

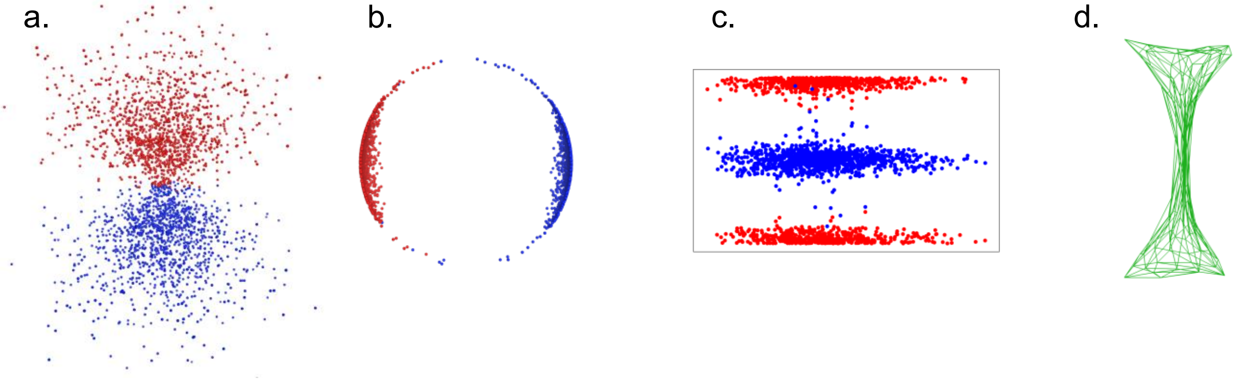

3. Size of frame set = 1250 datapoints

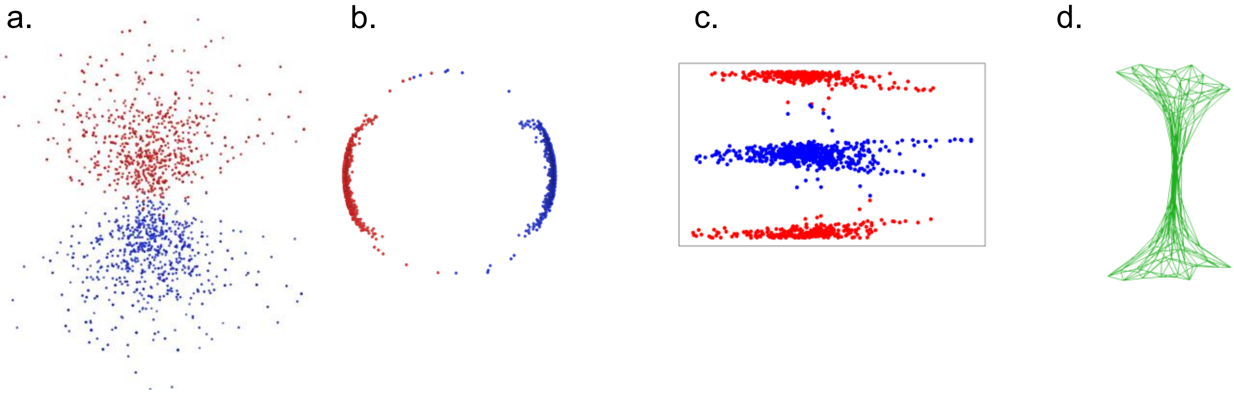

Application of SGTM on water HOMO frame Sets of Varying Sizes

1. Size of frame set = 620 datapoints

a.

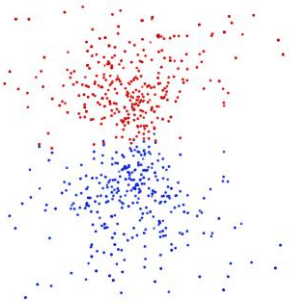

b.

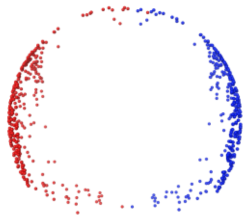

c.

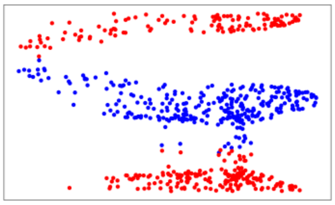

d.

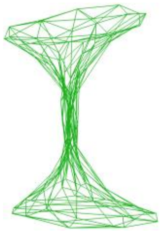

2. Size of frame set = 310 datapoints

a.

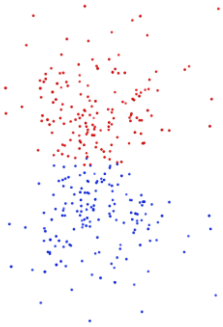

b.

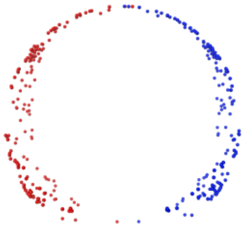

c.

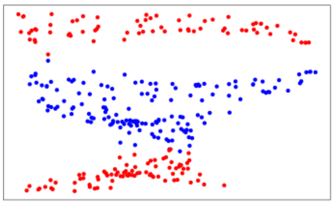

d.

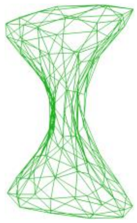

Application of SGTM on Tox21 database

Figure 15. Visualisation of compound distribution using (a) GTM, (b) SGTM projections, and (c) example of molecular structures of the cluster. Yellow points on the maps indicate selected compounds, which are shown below. Black points represent the remaining compounds from the Tox21 database.

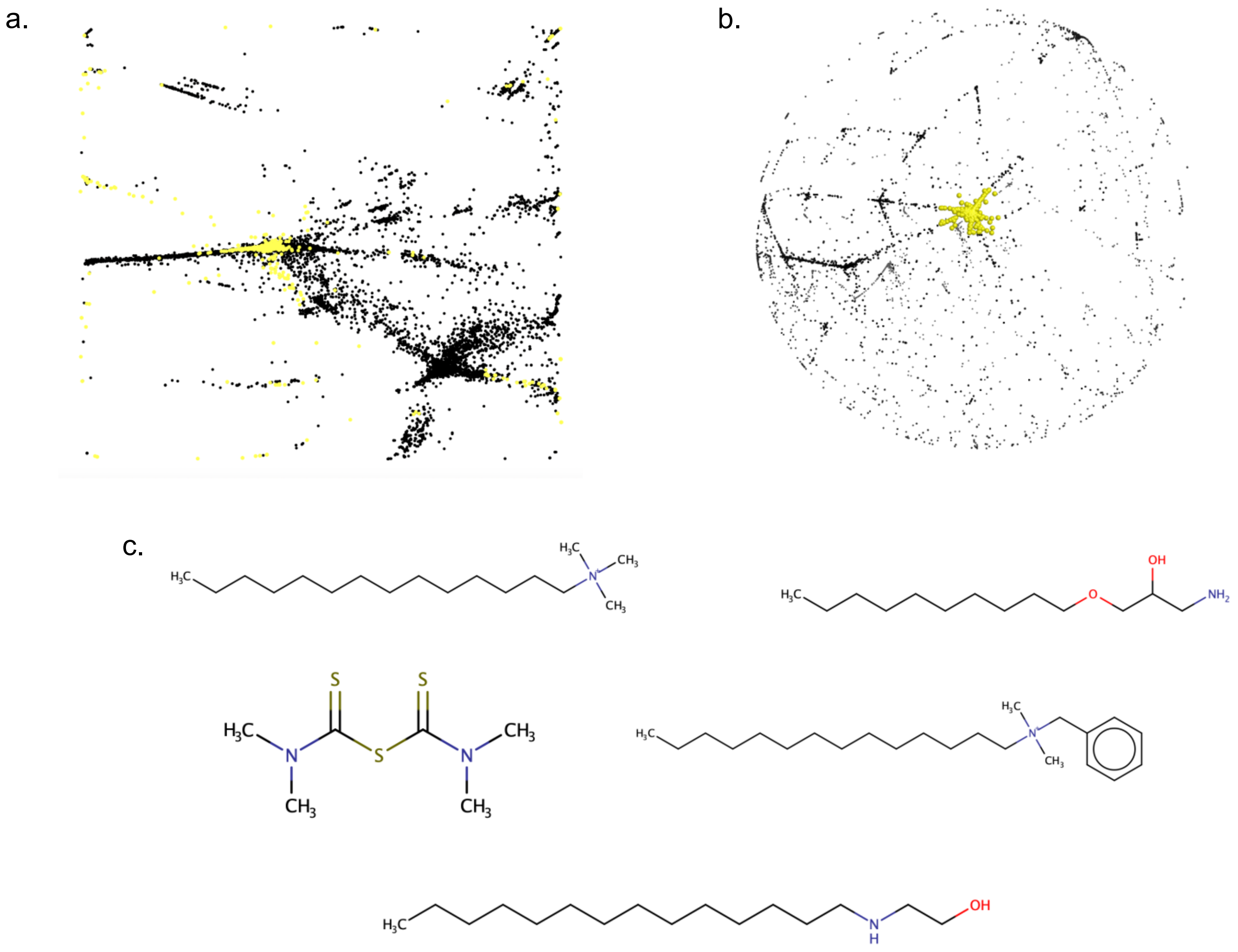

Richard, A. M.; Huang, R.; Waidyanatha, S.; Shinn, P.; Collins, B. J.; Thillainadarajah, I.; Grulke, C. M.; Williams, A. J.; Lougee, R. R.; Judson, R. S.; Houck, K. A.; Shobair, M.; Yang, C.; Rathman, J. F.; Yasgar, A.; Fitzpatrick, S. C.; Simeonov, A.; Thomas, R. S.; Crofton, K. M.; Paules, R. S.; Bucher, J. R.; Austin, C. P.; Kavlock, R. J.; Tice, R. R. The Tox21 10K Compound Library: Collaborative Chemistry Advancing Toxicology. *Chem. Res. Toxicol.* **2021**, *34* (2), 189–216. https://doi.org/10.1021/acs.chemrestox.0c00264.
